# Strategy of *Pseudomonas pseudoalcaligenes* C70 for effective degradation of phenol and salicylate

**DOI:** 10.1371/journal.pone.0173180

**Published:** 2017-03-03

**Authors:** Merike Jõesaar, Signe Viggor, Eeva Heinaru, Eve Naanuri, Maris Mehike, Ivo Leito, Ain Heinaru

**Affiliations:** 1 Institute of Molecular and Cell Biology, Faculty of Science and Technology, University of Tartu, Tartu, Estonia; 2 Institute of Chemistry, Faculty of Science and Technology, University of Tartu, Tartu, Estonia; MJP Rohilkhand University, INDIA

## Abstract

Phenol- and naphthalene-degrading indigenous *Pseudomonas pseudoalcaligenes* strain C70 has great potential for the bioremediation of polluted areas. It harbours two chromosomally located catechol *meta* pathways, one of which is structurally and phylogenetically very similar to the *Pseudomonas* sp. CF600 *dmp* operon and the other to the *P*. *stutzeri* AN10 *nah* lower operon. The key enzymes of the catechol *meta* pathway, catechol 2,3-dioxygenase (C23O) from strain C70, PheB and NahH, have an amino acid identity of 85%. The metabolic and regulatory phenotypes of the wild-type and the mutant strain C70Δ*pheB* lacking *pheB* were evaluated. qRT-PCR data showed that in C70, the expression of *pheB*- and *nahH*-encoded C23O was induced by phenol and salicylate, respectively. We demonstrate that strain C70 is more effective in the degradation of phenol and salicylate, especially at higher substrate concentrations, when these compounds are present as a mixture; i.e., when both pathways are expressed. Moreover, NahH is able to substitute for the deleted PheB in phenol degradation when salicylate is also present in the growth medium. The appearance of a yellow intermediate 2-hydroxymuconic semialdehyde was followed by the accumulation of catechol in salicylate-containing growth medium, and lower expression levels and specific activities of the C23O of the *sal* operon were detected. However, the excretion of the toxic intermediate catechol to the growth medium was avoided when the growth medium was supplemented with phenol, seemingly due to the contribution of the second *meta* pathway encoded by the *phe* genes.

## Introduction

In nature, aromatics are an extensively distributed class of organic compounds. Xenobiotic aromatic pollutants are usually the major concern because of their environmental persistence and toxicity to living organisms. Despite that, microorganisms possess biochemical pathways enabling them to use these compounds as sole carbon and energy sources [1–4].

Biodegradation pathways of aromatic compounds converge towards intermediates such as catechol, protocatechuate, gentisate and hydroquinone before ring cleavage occurs [5]. In aerobic degradation pathways, catechol and its substituted derivatives are channelled towards either an *ortho*-cleavage pathway by catechol 1,2-dioxygenase (C12O) or a *meta*-cleavage pathway by catechol 2,3-dioxygenase (C23O) [3, 5].

Extradiol dioxygenases (C23Os) are more versatile than the intradiol enzymes (C12Os), cleaving very different substrates and occurring in a wider variety of pathways [6]. It is vital for bacteria to avoid the accumulation of catechol during the degradation of aromatic compounds because it can repress the synthesis of the catechol dioxygenase systems [7–11]. For example, a high inhibitory effect of this metabolite was confirmed in *Pseudomonas putida* F1, where catechol concentrations higher than 0.09 mM significantly decreased both benzene- and catechol-associated respiration of the strain [11]. To avoid the accumulation of toxic intermediates, bacteria have developed multiple strategies: the expression of C23O-reactivating ferredoxins, the recruitment of inactivation-resistance C23Os, gene duplications that increase catechol consumption or the balanced induction of enzymes involved in the production and degradation of catechol [12, 13].

Several research groups have found that bacteria possess multiple C12Os and/or C23Os [14–20]. Gene duplication seems to be a widespread phenomenon amongst TOL plasmids and gives them a selective advantage in the natural environment over those with only single copies [16]. *P*. *aeruginosa* JI104, which can utilize different aromatic compounds, has three C23Os. These isoenzymes can coexist as a result of gene duplication and further divergence due to the accumulation of mutations in the duplicated genes that are free of selective pressures [21]. Possessing multiple catechol dioxygenases helps keep the intracellular concentration of catechol or it derivatives low, probably preventing its toxicity [20].

In the natural environment, bacteria are mostly exposed to mixtures of aromatic compounds, and their survival and distribution depends on the capability and versatility of bacterial metabolism [9, 22]. In the Baltic Sea, bacterioplankton is influenced by coastal industries and marine traffic [23], both of which can cause pollution containing various compounds. We have shown previously that bacteria isolated from this environment possess IncP-9 family plasmids [24] and can degrade phenol, benzoate, *m*-toluate, salicylate, naphthalene [25] and toluene [26]. Vedler *et al*. [25] also showed that 3 out of 38 strains degrading phenol via the catechol *meta* pathway have two to three phylogenetically different C23O genes. One of these strains, *Pseudomonas pseudoalcaligenes* C70, is able to grow on naphthalene in addition to phenol, but not on benzoate or toluate, and was selected for future investigation in this study. Whole genome sequencing revealed the occurrence of three genetically different sequences of the 16S rRNA gene, one most similar to *P*. *pseudoalcaligenes* and the other two to *P*. *mendocina*. Despite this finding, strain C70 was classified as a *P*. *pseudoalcaligenes* based on the sequence of the *rpoB* gene and its phenotypic characteristics [25].

The aim of the present research was to identify the physiological role of C23Os in *P*. *pseudoalcaligenes* strain C70 and to evaluate their potential to degrade a mixture of phenol and salicylate. We would like to note that bacteria endowed with functional lower *nah* and *phe* (*dmp*-like) pathways have never been described and published. We characterized the expression and functioning of the redundant C23O genes (*pheB* and *nahH*) by generating the C70Δ*pheB* strain and by analysing the effect of this mutation on cell growth and the effective consumption of a mixture of salicylate and phenol.

## Materials and methods

### Bacterial strains, plasmids and culture conditions

The studied *Pseudomonas pseudoalcaligenes* strain C70 [24, 25] (CELMS, University of Tartu, Estonia) and the other strains and plasmids are shown in S1 Table. Pure cultures were stored in 30% glycerol at -80°C. The C70 strain was incubated on agar plates with minimal medium containing M9 salts [27] and trace elements [28] supplemented with phenol (PHE) (1.3 mM), salicylate (SAL) (1.3 mM) or R2A medium (Difco, USA) at 30°C. Strain C70Δ*pheB* was grown on LB medium or naphthalene vapour with kanamycin (Km, 50 μg ml^-1^) at 30°C. *Escherichia coli* strain DH5α containing the pTZ57R/T plasmid (Thermo Fisher Scientific, USA) was grown on LB medium with ampicillin (15 μg ml^-1^) at 37°C. Batch cultivation of cells was performed in 200 ml Erlenmeyer flasks containing 50 ml minimal medium supplemented with 3 mM PHE and SAL as a single substrate and as a mixture at 30°C on a rotary shaker. Growth was followed spectrophotometrically at 580 nm.

### Sequencing and assembly of the draft genome of strain C70

The total DNA from the bacterial strain was isolated using the QIAGEN Plasmid Mega Kit (QIAGEN, Netherlands) that enriches the plasmid DNA over the chromosomal DNA. The isolated DNA was fragmented, samples for whole genome sequencing were prepared using the Illumina TruSeq® DNA PCR-Free LT Sample Preparation Kit (Illumina, USA) and were sequenced by default paired-end read protocols (2x100 bp) using an Illumina HiSeq2500 (Illumina, USA).

The whole genome was assembled using the Velvet version 1.2.09 software [29] as described previously [26]. The total length of the assembled contigs was 4,561,427 bp (576-fold sequencing coverage) in 506 contigs. The contig N50 was 549,228 bp with the longest scaffold (1,109,403 bp).

Gaps in *nah* lower and *ph*e operons were sequenced using the 3730*xl* DNA Analyzer (Applied Biosystems, Thermo Fisher Scientific, USA) using the BigDye® Terminator v3.1 Cycle Sequencing Kit (Applied Biosystems, Thermo Fisher Scientific, USA) and protocols provided by the manufacturer.

The nucleotide sequences of the *nah* lower and *phe* operons of the strain C70 were deposited in GenBank under accession numbers KU695544 and KU695543, respectively.

### DNA analysis

The GenBank database search used the NCBI ORF Finder and BLAST programs. The deduced amino acid sequences of C23Os of strain C70 were aligned with C23Os of the reference strains from the GenBank database using BioEdit version 7.0.5.3 [30]. Program MEGA6 was applied to construct the phylogenetic tree. Next to the branches is the percentage of replicate trees in which the associated taxa clustered together in the bootstrap test (1000 replicates) [31]. The tree is drawn to scale, with branch lengths in the same units as those of the evolutionary distances used to infer the phylogenetic tree. The JTT matrix-based method was used for computing the evolutionary distances that are presented as the units of the number of amino acid substitutions per site [31].

### qRT-PCR

Total RNA for quantification of mRNA levels of *nahH* and *pheB* genes was isolated from exponential phase cells of *P*. *pseudoalcaligenes* C70 using the NucleoSpin RNA II kit (Macherey-Nagel, USA). Cells were grown on R2A medium or R2A and 2.5 mM SAL, R2A and 2.5 mM PHE or R2A and the mixture of PHE and SAL (both 2.5 mM) media. DNase I (Thermo Fisher Scientific, USA) was used to treat the RNA samples. A NanoDrop ND-1000 Spectrophotometer (Thermo Fisher Scientific, USA) was used to assess the RNA concentrations and purity. The SuperScript III Platinum SYBR green one-step qRT-PCR kit (Invitrogen, Thermo Fisher Scientific, USA) was used according to the manufacturer’s protocol (in a total reaction volume of 10 μl) for the quantitative reverse transcription-PCR (qRT-PCR) assay performed on the Rotor-Gene Q system (QIAGEN). A 10 ng portion of the total RNA was used for each reaction. The *nahH* and *pheB* genes were amplified using the primers C70aF and C70aR, and C70bF and C70bR, and the *polA* gene was amplified using the primers polAXhoylev and polAXhoall (S2 Table). The PCRs were performed using the following program: 50°C for 3 min and 95°C for 5 min, followed by 40 cycles of denaturation for 15 s, annealing at 58°C for 30 s, extension at 72°C for 20 s and 40°C for 1 min. At the end of the run, melting curve analyses were performed by increasing the temperature from 72 to 95°C and 3 s at 0.35°C with continuous fluorescence recording. The Rotor-GeneQ software version 2.02 (QIAGEN) was used to analyse the raw data, and mRNA amounts were calculated using the LinRegPCR software version 2013.0 [32]. Data from three separate qRT-PCR experiments performed on three independently extracted RNAs were averaged and normalized against *polA* levels.

### Enzyme activity assay

Crude extracts were prepared and the activities of C23O and the protein concentrations were measured as described in [33] and [34]. The accumulation of 2-hydroxymuconic semialdehyde (HMS) was monitored by measuring the absorbance at 375 nm (ɛ = 14.7 mM l^-1^ cm^-1^).

### Mutagenesis of the putative C23O genes

The bacterial strains and plasmids used and constructed in the *nahH* and *pheB* mutagenesis experiment are described in S1 Table. To disrupt the *nahH* and *pheB* genes in *P*. *pseudoalcaligenes* strain C70, a 1.8 kb *nahH*-containing DNA region and a 1.8 kb *pheB*-containing DNA region were amplified by PCR from genomic DNA with the corresponding pairs of primers (S2 Table) C70a1F –C70a2R and C70b1F –C70b2R. The amplified PCR products were cloned into the pTZ57R/T vector (Thermo Fisher Scientific, USA), resulting in pTZ57R/C70nahH and pTZ57R/C70pheB constructs. Then, the *nahH* region was excised from pTZ57R/C70nahH with the *Aat*II and *Not*I enzymes, and *pheB* was excised from pTZ57R/C70pheB using *Eco130*I. Approximately 1 kb sized DNA fragments of both genes, *nahH* and *pheB*, were deleted and replaced with the Gm^r^ and Km^r^ gene, respectively, which were earlier amplified by PCR from the pBK-miniTn7-ΩGm using the primers GmY and GmA (S2 Table) and from pUTmini-Tn5 Km2 with primer KmSac (S2 Table) and afterwards cleaved with *Ecl136*II. Before ligation of the Gm^r^ and Km^r^ gene fragments into the open constructs, the *Aat*II, *Not*I and *Eco130*I ends of the constructs were blunt-ended. From the pTZ57RΔC70nahH::gm and pTZ57RΔC70pheB::km constructs, the sequences ΔC70nahH::gm and ΔC70pheB::km were excised with *Kpn*I-*Pae*I, respectively, and inserted into the plasmid vector pGP704 to obtain the constructs pGP704ΔC70nahH::gm and pGP704ΔC70pheB::km. These constructs were transferred from *E*. *coli* CC118λpir (S1 Table) into the *P*. *pseudoalcaligenes* strain C70 by conjugation using the helper plasmid pRK2013 (S1 Table). Transconjugants were chosen on agar plates supplemented with kanamycin (50 μg ml^-1^) and naphthalene vapour or gentamycin (10 μg ml^-1^) and PHE; benzylpenicillin (1,500 μg ml^-1^). Transconjugants were confirmed with PCR analysis using the primers KmOc (S2 Table) and C70b1F and GmA and C70a1F, and with *nahH* and *pheB* inner primers C70aF and C70aR; C70bF and C70bR (S2 Table). Conjugation experiments were carried out in triplicate.

### Measurement of phenol and salicylate tolerance using respiration analysis

Oxygen-consumption rates (μ) of the bacterial cells pre-grown on 1.3 mM PHE and SAL were determined using the manometric respiratory system OxiTop® Control sensor (WTW, Germany). Tests were performed in two replicates in 250 ml bottles containing 100 ml mineral medium [27] with different PHE (0.7–15 mM) and SAL (0.7–8 mM) concentrations at 30°C for up to five days. The initial cell concentration in the bottles was 10^6^ CFU ml^-1^ (*wt* C70 and C70Δ*pheB*). Oxygen consumption was measured based on the pressure drop in the bottles [35], and calculations of the specific oxygen-consumption rate and parameters of inhibition of the specific oxygen-consumption rate by PHE and SAL as determined using the Luong equation were performed as described by [36].

### Chemical analyses

All samples taken from growth experiments for chemical analysis were centrifuged at 12,000 x g for 1 min at 4°C, and the supernatants were stored at -20°C until analysis. Analysis of the substrates and metabolites were performed using an Agilent Technologies (USA) 1200 Series high-performance liquid chromatography (HPLC) system with a Quaternary pump, an Autosampler and a 5-channel variable wavelength UV-Vis detector. The separation of compounds was achieved using an Agilent ZORBAX Eclipse XDB-C18 (4.6 x 250 mm i.d., 5 μm) analytical column and guard column (12.5 x 4.6 mm i.d.) of the same material (Agilent Technologies, USA). Elution was carried out at a flow rate of 0.80 ml min^-1^ within 15 minutes with a mobile phase gradient as follows: 0 min B (methanol)/C (standard buffer of 0.1% formic acid and 1 mM ammonium acetate, pH ~2.8) 45/55, 9 min 75/25, 11 min 45/55, and 13 min 45/55. The separation of the mixtures (injection volume 20 μl) was carried out at room temperature and the UV-Vis detector was set at 274 nm. The combined standard uncertainties of the results were between 2.1% and 3.4%. The preparation of the sample, instrumental measurements (both samples and calibration standards) and integration of the peaks are the main contributions to the uncertainties [37].

## Results and discussion

### Analysis of the genes of the phenol and naphthalene (lower) catabolic pathways

**Fig 1:**
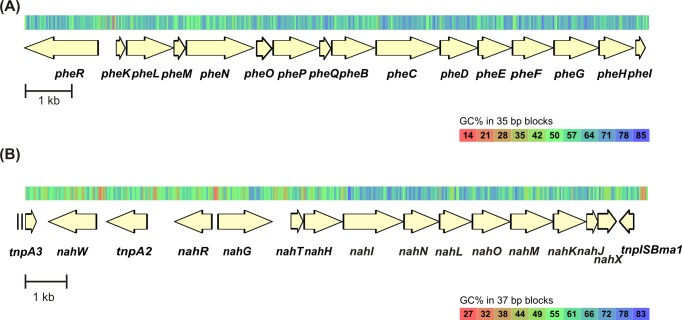
Genetic organization of the *phe* and *sal* operons of *Pseudomonas pseudoalcaligenes* C70. (A) The genes of the *phe* operon—*pheR*, transcriptional regulator: *pheKLMNOP*, components of phenol hydroxylase; *pheQ*, ferredoxin-like gene; *pheB*, catechol 2,3-dioxygenase; *pheC*, 2-hydroxymuconic semialdehyde dehydrogenase; *pheD*, 2-hydroxymuconic semialdehyde hydrolase; *pheE*, 2-oxopent-4-dienoate hydratase; *pheF*, acetaldehyde dehydrogenase; *pheG*, 4-hydroxy-2-oxovalerate aldolase; *pheH*, 4-oxalocrotonate decarboxylase; *pheI*, 4-oxalocrotonate isomerase. (B) The genes of the *sal* operon—*tnpA*, transposase-like gene: *nahW*, salicylate 1-hydroxylase; *nahR*, regulatory gene; *nahG*, salicylate 1-hydroxylase; *nahT*, chloroplast ferredoxin-like protein coding gene; *nahH*, catechol 2,3-dioxygenase; *nahI*, hydroxymuconic semialdehyde dehydrogenase; *nahN*, hydroxymuconic semialdehyde hydrolase; *nahL*, 2-oxopent-4-enoate hydratase; *nahO*, acetaldehyde dehydrogenase; *nahM*, 2-oxo-4-hydroxypentanoate aldolase; *nahK*, 4-oxalocrotonate decarboxylase; *nahJ*, 4-oxalocrotonate isomerase; *nahX*, hypothetical protein coding gene; *tnpISBma1*, transposase-like gene.

*Pseudomonas pseudoalcaligenes* strain C70 has two catechol *meta* pathways for the degradation of phenol and naphthalene (Fig 1). One of them is a part of the phenol degradation operon *pheRKLMNOPQBCDEFGHI*, which is structurally similar to the *dmp* operon of *Pseudomonas* sp. CF600. This operon is regulated by the XylR family-type regulatory gene *pheR* [38]. The other catechol *meta* route is a naphthalene lower pathway encoded by the classical *nah* operon (*nahGTHINLOMKJX*, also known as the *sal* operon) as described in *P*. *putida* G7 pNAH7 [39]. These enzymes convert salicylate over catechol to pyruvate and acetyl-CoA. This *nah* operon is regulated by the LysR family-type regulatory gene *nahR* [39]. The analysis of the *sal* operon structure in *P*. *pseudoalcaligenes* strain C70 revealed its high identity level with the *sal* operon described in *P*. *stutzeri* AN10 (CCUG 29243) [40]. Both strains have a second salicylate 1-hydroxylase gene (named as *nahW* in *P*. *stutzeri* AN10), in addition to *nahG* described from strain NAH7, and they share 100% amino acid identity. The strains NAH7, C70 and AN10 have the *nahX* gene with unknown function in the *sal* operon [41].

As the *nah* lower operon is located between two transposable elements in C70, we can hypothesize that this operon was probably acquired from an outside source through horizontal gene transfer. Operons that encode aromatic degradation enzymes located on plasmids or chromosomes are frequently carried on transposons or flanked by insertion elements, providing their transferability [42]. Amino acid similarities of the different aromatic pathway enzymes and the diverging organization of catabolic genes suggest that several different gene clusters (e.g., *meta* pathways) may be combined in modules to which other peripheral genes may be added. Furthermore, it shows that many DNA rearrangements have occurred during the evolution of different pathways. This is also supported by the finding that the exposure of a community to a severe environment induces gene transfer, recombination and transposition events, thus conferring a higher survival capacity [5].

The strain C70 was shown to contain one large plasmid; therefore, the total DNA for whole genome sequencing was isolated using a method that enriches plasmid DNA over the chromosomal DNA. This led to some assembled contigs with at least 10-fold higher coverage, suggesting that these contigs are most likely of plasmid origin. However, all the genes necessary for phenol and naphthalene degradation were found on contigs with lower coverage, indicating that the *phe* and *sal* operon are located in the chromosome. In the plasmid, we did not find any aromatic catabolic genes. The *nahH* gene has been considered a plasmid encoded in the genus *Pseudomonas*, but in most of the naphthalene-degrading *Pseudomonas stutzeri* strains studied to date, this gene is chromosomally encoded [43]. The analysis of sequences did not reveal any catechol 1,2-dioxygenase or gentisate 1,2-dioxygenase encoding genes that might also be involved in the degradation of PHE and SAL. Because there were few data suggesting the coexistence of two catechol *meta* pathways in the same strain, we focused on the characterization of the key enzymes C23Os.

### Phylogeny of redundant catechol 2,3-dioxygenases of strain C70

For phylogenetic analysis, a neighbour-joining tree based on deduced amino acid sequences of C23Os (PheB and NahH, 307 aa) of C70 was constructed together with C23O proteins accessible in GenBank (S1 Fig). Our analysis indicated that PheB from the *phe* operon in strain C70 was closest to the PhhB from *P*. *putida* P35X, with an amino acid identity of 95% (Cluster II in S1 Fig). As expected, NahH from strain C70 clustered together with the other C23O genes determined by *nah* lower operons (Cluster I, S1 Fig) and it was closest to chromosomally encoded NahH from *P*. *stutzeri* AN10, with an amino acid identity of 99.7%. NahH from C70 differed from that of AN10 by two bases, resulting in a tyrosine (Tyr) at position 218 instead of histidine (His). Two C23Os of strain C70, PheB and NahH, shared an amino acid identity of 85%. They also differed at position 218; NahH had a Tyr in this position, but PheB had phenylalanine (Phe). The catalytic properties of C23Os can change drastically even due to the change of a single amino acid [44]. Pseudomonads (for example, strain 1YB2) that have C23Os with a Tyr at amino acid position 218 are found in contaminated environments with high and low concentrations of benzene and toluene, which is compatible with a low turnover number and a high affinity of C23O for catechol. However, a C23O carrying His at position 218 (as in the case of the *Pseudomonas* sp. strain 1YdBTEX2) could be isolated only from a site with high concentrations of the same compounds, which is in accordance with a high turnover number and a low affinity for catechol. This suggests that in response to pollutant concentrations, the enzymes were positively selected in the environment [44, 45].

Our studied strain was isolated from Baltic Sea water taken from the southern area of the Finnish gulf, near a high sea traffic and port area; therefore, the water may be slightly contaminated with aromatic compounds. *P*. *stutzeri* AN10 was isolated from polluted marine sediments of the West Mediterranean Sea [46].

### Construction of mutant strains and testing their tolerance to higher substrate concentrations

To establish the role of the redundant catechol *meta* pathways in strain C70, the knockout mutants of the two genes *pheB* and *nahH*, encoding the key enzymes of both pathways, C23O, were constructed. While the mutant strain C70Δ*pheB* (with the *pheB* gene disrupted with Km^r^ gene) was successfully obtained, we were unable to construct C70Δ*nahH*, although we attempted to use the alternative method of Martinez-Garcia and de Lorenzo [47].

**Fig 2:**
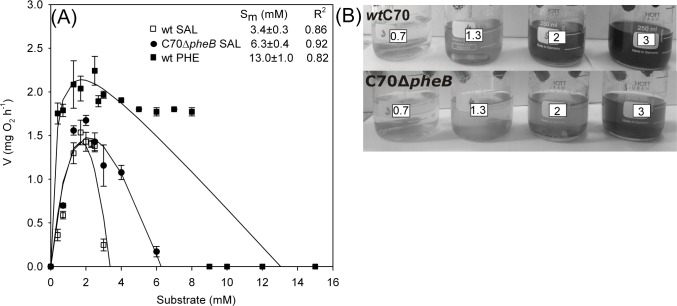
Determination of maximum inhibitory concentrations of phenol and salicylate, and accumulation of intermediates in salicylate-containing growth medium. (A) The phenol (PHE)- and salicylate (SAL)-dependent oxygen consumption rate curves of *wt* and C70Δ*pheB* strains for determination of the maximum inhibitory concentrations of phenol and salicylate, Sm, using the Luong model. Error bars indicate the standard deviation. (B) Accumulation of dark brown metabolic intermediates of SAL catabolism with different SAL concentrations (0.7, 1.3, 2.0 and 3.0 mM) in *wt* and C70Δ*pheB* strains.

The higher concentrations of aromatic compounds and their metabolites in the growth medium can be toxic to bacteria. For example, the growth of *Escherichia coli* and *Burkholderia cepacia* were inhibited in LB by the addition of 5 mM SAL [48] and on phenanthrene by SAL at concentrations >1.25 mM [49], respectively. The tolerance of *wt* C70 and C70Δ*pheB* strains to the high concentration of PHE and SAL was tested by determination of the inhibition of the oxygen consumption rate (Fig 2).

The C70Δ*pheB* strain tolerated higher SAL concentrations than the *wt*. The maximum inhibitory concentrations of SAL (at which no oxygen consumption was observed, *S*_*m*_, using the Luong model) were 6.3±0.4 mM for C70Δ*pheB* and 3.4±0.3 mM for the *wt* strain. During growth on higher concentrations of SAL, the formation of dark pigments was observed at concentrations of 2 mM and 3 mM in the *wt* and C70Δ*pheB* strains, respectively (Fig 2B). These pigments are the result of the polymeric aggregates of quinonic intermediates, spontaneously formed in solution after the oxidation of catechol, and this dark brown pigment is toxic even for bacteria able to degrade catechol [20, 50]. A highly inhibitory effect of accumulating catechol to benzene-associated respiration has been shown in *Pseudomonas putida* F1 [11]. The maximum inhibitory concentration (*S*_*m*_) of PHE in the *wt* strain was 13.0±1.0 mM and the accumulation of insoluble dark pigments in the growth medium was not determined. Juang and Tsai [10] have also shown that the inhibitory effect of SAL on the growth of *Pseudomonas putida* is higher than that of PHE.

Based on these data, we can conclude that the maximum inhibitory concentration of SAL in the strain possessing two catechol *meta* pathways and the C70Δ*pheB* strain, with a disrupted *pheB* gene and an intact *nahH* gene, are different. At the same time, the SAL concentrations where the values of the maximum specific oxygen-consumption rate were the highest are quite similar. In addition, the *wt* strain can tolerate higher PHE concentrations than SAL concentrations.

### Effect of inducers on the expression and activity of two catechol 2,3-dioxygenase genes

**Fig 3:**
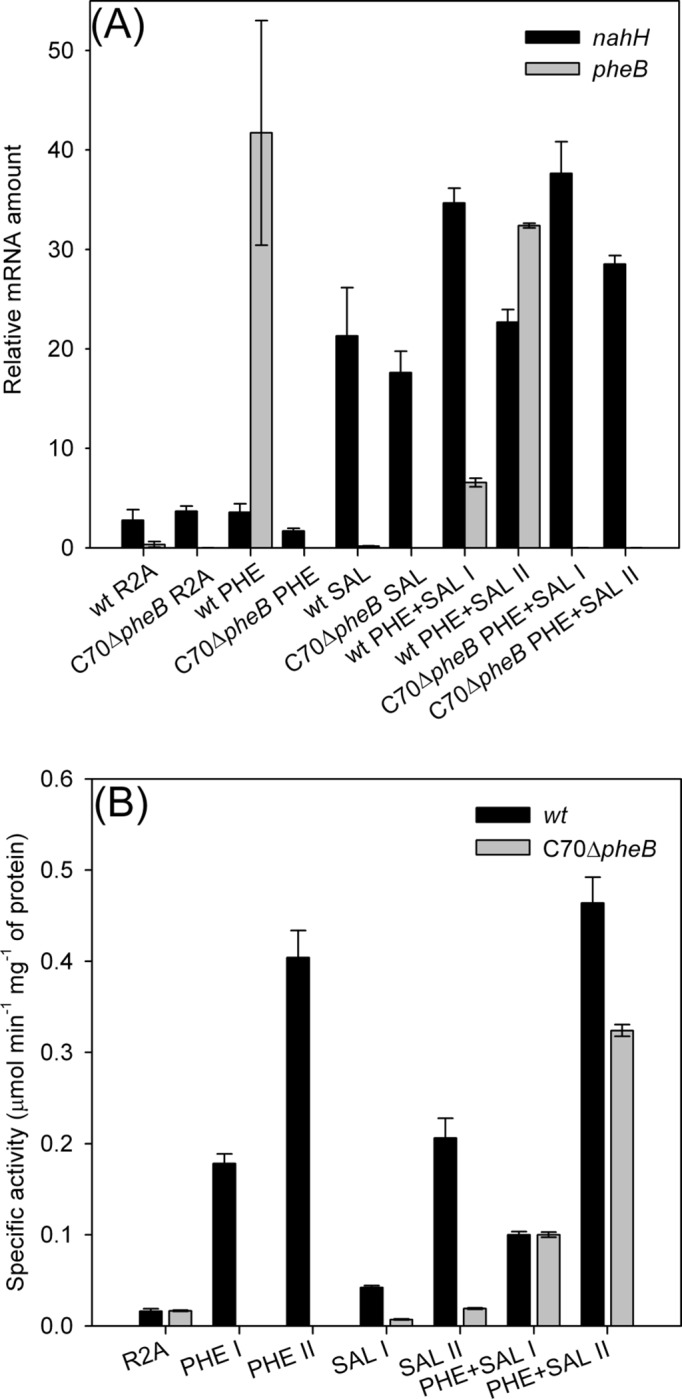
Expression levels of *nahH* and *pheB* and specific activities of C23O in strains C70 and C70Δ*pheB* on different growth substrates. (A) For qRT-PCR analysis of the mRNA transcripts, the cells were grown on R2A medium, R2A and phenol (PHE, 2.5 mM), R2A and salicylate (SAL, 2.5 mM), or R2A and the mixture of SAL and PHE. The relative expression ratios (y-axis) were calculated as relative values of the reference gene *polA* expression level of the respective C70 strain. (B) Specific activities of C23O of the *wt* and C70Δ*pheB* strains were determined from cells grown on minimal medium supplemented with phenol, salicylate or the mixture of them (all substrates 3 mM). Samples were taken from the middle (I or no specification) or the late-exponential (II) growth phase.

The induction experiments were carried out to determine the effect of PHE and SAL as inducers on the expression of C23Os in *wt* and C70Δ*pheB*. The bacterial cells were grown to mid-log phase on R2A or on a mixture of R2A and SAL (2.5 mM) or/and PHE (2.5 mM), after which the corresponding mRNA was quantified by quantitative reverse transcription-PCR. The expression levels of the *pheB* and *nahH* genes are presented as relative to the housekeeping reference gene *polA* in Fig 3A. The results revealed that the expression of C23O was achieved by the induction of *pheB* by PHE and *nahH* by SAL in *wt* C70. In C70Δ*pheB*, the expression level of the *nahH* gene induced by SAL remained at the same level as in the *wt* strain; in the case of growth on PHE, the expression level of the *nahH* gene remained at the basal level (Fig 3A). As expected, the disrupted *pheB* gene was not expressed in C70Δ*pheB*. In addition, it seems that NahH is induced by SAL, although there is some basal level of expression without SAL, whereas PheB is inducible only by PHE (Fig 3A).

Specific activity values of C23O determined in the *wt* strain grown on minimal medium containing PHE or SAL (Fig 3B) supported the results obtained in the qRT-PCR experiments (Fig 3A) that the activity of C23O is lower in cells grown on SAL than on PHE. At the same time, in C70Δ*pheB* grown in SAL, the specific activities were lower than that of the *wt* strain under the same conditions. The finding that specific activities are higher in the late-exponential than in the middle-exponential growth phase are in accordance with results obtained by Hugouvieux-Cotte-Pattat *et al*. [51] during the study of the induction of the TOL catabolic operons.

Since the expression levels and specific activities of C23Os in *wt* and C70Δ*pheB* strains on single substrates were different, experiments were conducted to clarify the degradation of SAL and PHE (both as a mixture and single substrate) by the studied strains.

### Growth on single substrates: Identification of accumulation of intermediates

**Fig 4:**
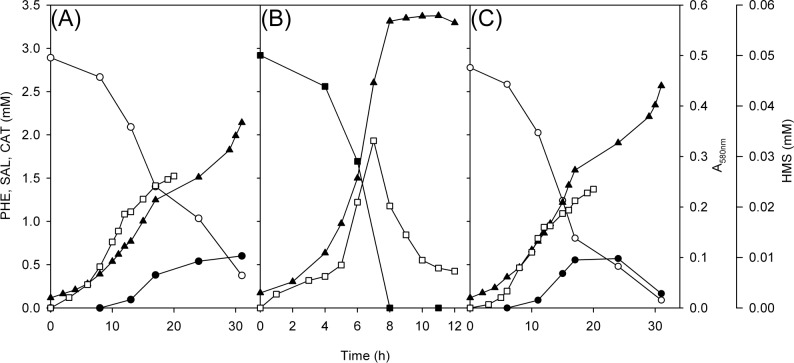
Growth, degradation of phenol and salicylate as single substrates and the accumulation of intermediates in strains C70 and C70Δ*pheB*. Growth (▲, measured as absorbance at 580 nm), degradation of phenol (■) and salicylate (○) (both 3 mM, as single substrates), and accumulation of catechol (●) and 2-hydroxymuconic semialdehyde (HMS, □) in strains C70 (A, B) and C70Δ*pheB* (C). Concentrations of the substrates and intermediates are the averages of triplicate determinations; the combined standard uncertainties of the results were between 2.1% and 3.4%.

As mentioned above, the accumulation of dark brown pigments was observed when strains were grown on SAL (3 mM) (Fig 2B). In addition, the accumulation of a yellow compound was observed in all experiments. Furthermore, the biomass yield of *wt* and C70Δ*pheB* grown on SAL (Fig 4A and 4C) was lower than that of the *wt* strain grown on PHE (Fig 4B). To determine which intermediates accumulate in the growth media, HPLC and spectrophotometric analysis was conducted.

Accumulation of 2-hydroxymuconic semialdehyde (HMS) was observed when strains were grown on SAL and PHE (Fig 4). However, catechol (CAT) was determined in the growth medium only during the growth of the *wt* and C70Δ*pheB* strains on SAL (Fig 4A and 4C) after the appearance of HMS. Based on these results, we hypothesized that NahH is more sensitive to the accumulation of HMS than PheB, and therefore, CAT accumulates when strains are grown on SAL. Consequently, a lower growth yield on SAL than on PHE was determined. These assumptions are supported by higher specific activity values (both in the middle and at the end of the logarithmic growth phase, Fig 3B) and expression levels of C23O in the *wt* strain grown on PHE rather than on SAL (Fig 3A). Although in the case of the growth of the C70Δ*pheB* strain on SAL a reversible accumulation of catechol was observed (Fig 4C), we cannot conclude whether it was consumed by the bacteria or if the concentration was diminished due to autoxidation. The formation of dark brown pigments after ~15 h of incubation on SAL caused an increase in optical density at 580 nm that interfered with the determination of biomass concentration at that wavelength.

The assumption that catechols have an inhibitory effect on C23Os has been confirmed by several groups [11, 50, 52], but the role of HMS in the degradation of aromatic compounds has received little attention. Muñoz *et al*. [11] reported that the accumulation of catechol has a highly inhibitory effect on benzene- and catechol-associated respiration. They also suggested that the accumulation of the yellow intermediate had an effect, but they did not investigate it further. While modelling the growth of *Pseudomonas* sp. NCIMB 9688 on benzene, Monero *et al*. [53] concluded that HMS is a competitive inhibitor and its accumulation within the cell has an effect on the kinetic control of the *meta* pathway by the enzyme responsible for HMS degradation. The formed HMS can be degraded in two branches of the *meta* pathway by 2-hydroxymuconic semialdehyde dehydrogenase (HMSD) or hydrolase (HMSH), and the primary substrates control the whole set of enzymes [3, 54, 55]. However, experiments in mutant strains have shown that HMS is preferentially degraded by HMSD [38, 55]. Our experiments indicated that the accumulation of HMS influences the expression levels and specific activities of the C23O of the *sal* operon when strains are cultivated on SAL as a single substrate. In the case of strain C70, the accumulation of catechol is observed after the appearance of HMS in the growth medium, resulting in an inhibition of C23O and in turn the inability to degrade all the catechol produced by two salicylate 1-hydroxylases.

Catechol degradation intermediates are also able to inactivate the dioxygenases responsible for their cleavage [12, 52, 56]. Cerdan *et al*. [57] have shown that sensitivity of different C23Os to catechols can vary. They reported that NahH from *P*. *putida* G7 has a higher affinity constant and apparent rate constant of enzyme inactivation for catechol and 3-methylcatechol than XylE from *P*. *putida* KT2440. To avoid the accumulation of toxic intermediates, bacteria have evolved different strategies. For example, a mutant strain of *P*. *putida* F1 gained the ability to degrade styrene due to a single base pair mutation in toluene dioxygenase that resulted in attenuated activity of the enzyme and decreased the rate of 3-vinylcatechol production compared to the *wt* strain [52]. Another possibility to avoid catechol toxicity is to increase the rate of catechol-consuming reactions through duplication of catechol dioxygenase genes. For example, *Cupriavidus pinatubonensis* JMP134 (pJP4) (formerly *Ralstonia eutropha* JMP134) requires multiple copies of chlorocatechol 1,2-dioxygenase for efficient turnover of 3-chlorocatechol and growth on 3-chlorobenzoic acid [56]. Therefore, the ability to grow on aromatic compounds depends on a delicate balance between catechol-producing and catechol-consuming reactions.

However, in the case of our strain, simply having two copies of the C23O gene in the genome does not have such an effect when grown on a single substrate. During the growth of the *wt* strain on SAL, the accumulation of catechol is still detected because only one gene coding for C23O is expressed.

### Growth on a mixture of substrates: Constitutively expressed *nahH* compensates for the function of disrupted *pheB*

**Fig 5:**
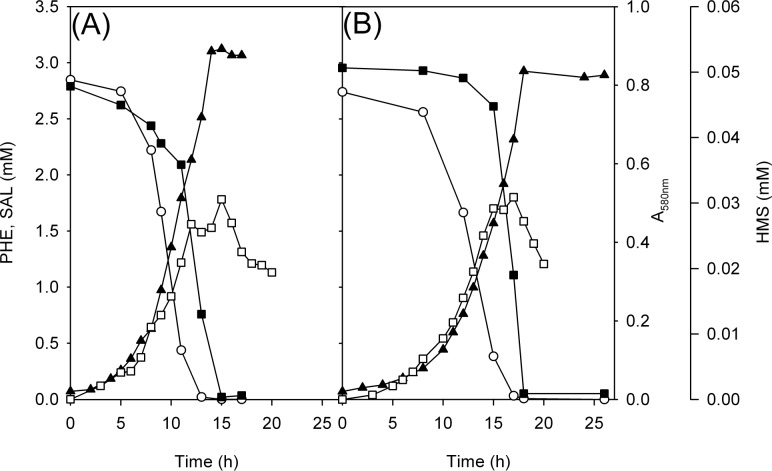
Growth and degradation of a mixture of phenol and salicylate and accumulation of intermediates in strains C70 and C70Δ*pheB*. Growth (▲, measured as absorbance at 580 nm), degradation of phenol (■) and salicylate (○) (both 3 mM), and accumulation of 2-hydroxymuconic semialdehyde (HMS, □) in strains C70 (A) and C70Δ*pheB* (B). The concentrations of the substrates are the averages of triplicate determinations; the combined standard uncertainties of the results were between 2.1% and 3.4%.

The strain C70Δ*pheB* is not able to grow on minimal medium containing PHE as a single substrate. However, we were curious about what would occur when grown on a mixture of PHE and SAL. The growth yield of *wt* and C70Δ*pheB* strains grown on the mixture of substrates was at the same level, although there was a delay in the growth of the C70Δ*pheB* strain compared with the *wt* strain (Fig 5).

HPLC analysis of the growth medium of both strains revealed that SAL was degraded before PHE, despite the fact that this substrate supports less efficient growth than PHE. This is probably due to the presence of two salicylate hydroxylase genes. Additionally, the accumulation of catechol was not detected (Fig 5). qRT-PCR analysis of the expression of *nahH* and *pheB* genes from *wt* and C70Δ*pheB* growing on R2A with a mixture of substrates showed that the *nahH* gene was expressed at a high level in the middle-exponential growth phase, whereas the *pheB* gene expression in the *wt* was higher in the late-exponential growth phase (Fig 3A). These data are in accordance with the results of the determination of specific activities of C23O, where higher values were also observed in the late-exponential phase (Fig 3B). The expression level of the *nahH* in C70Δ*pheB* grown on a mixture of PHE and SAL is almost at the same level through the exponential growth phase, although higher C23O activity values were obtained from late-exponential growth phase cells (Fig 3). Based on these results, we suggest that *nahH* can substitute/complement the *pheB* gene while strains are grown in a mixture of substrates, allowing the C70Δ*pheB* to use PHE as the growth substrate (Fig 5). Furthermore, as catechol does not accumulate in *wt* or C70Δ*pheB* in conditions where the *meta* pathway encoded by the *phe* genes is also expressed, the enzymes of this pathway downstream from C23O probably also contribute to the degradation of CAT and HMS produced from SAL.

Results obtained from the laboratory experiments with single strains and single substrates are usually not applicable in real pollution situations, as often the mixture of compounds is exposed to nature and the degradation of a particular component can be affected by other factors such as formed intermediates [7–9, 58]. In the case of strain C70, we found that this strain benefits from the two redundant catechol *meta* pathways, resulting in more efficient biodegradation of SAL only when PHE is also present in the growth medium and both pathways are expressed.

**Fig 6:**
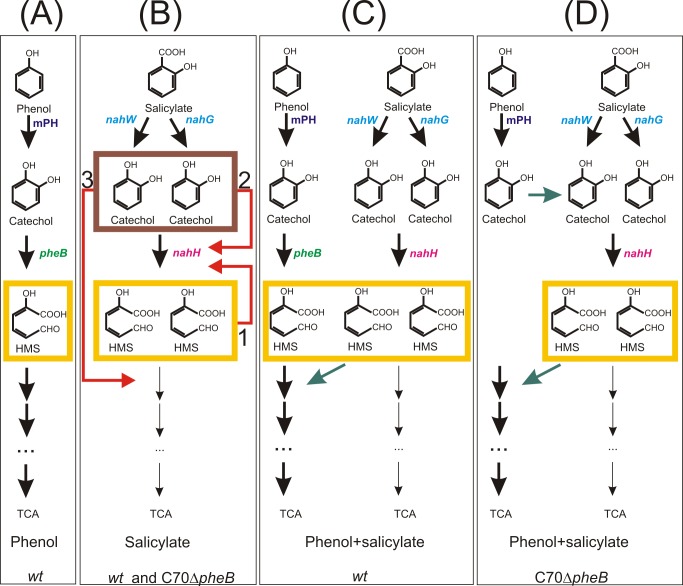
Role of accumulation of intermediates in the catabolism of phenol and salicylate as single or mixed substrates in C70 and C70Δ*pheB*. The figure sketches the degradation pathways and genes (mPH—multicomponent phenol hydroxylase; *pheB* and *nahH*—C23O; *nahW* and *nahG*—salicylate 1-hydroxylase) involved in phenol and salicylate catabolism as single (A, B) and mixed substrates (C, D). The supposed negative effect of accumulating intermediates 2-hydroxymuconic semialdehyde (HMS, yellow box) and catechol (brown box) to the functioning of involved catabolic genes are indicated with red arrows (with numbers 1, 2 and 3). Green arrows point to convergence or divergence of degradation of intermediates.

To sum up, we demonstrate that *P*. *pseudoalcaligenes* strain C70, harbouring two catechol *meta* pathways, encoded by two redundant catabolic operons, *phe* and *nah*, is more effective in the degradation of PHE and SAL, especially at higher substrate concentrations, when these compounds are present as a mixture; i.e., when both pathways are expressed. Induction experiments showed that NahH can substitute/complement the deleted PheB in PHE degradation when strains are cultivated in the mixture of substrates (Fig 6D). The accumulation of yellow HMS was observed in all substrates while the toxic intermediate catechol was detected in the growth medium only during the degradation of SAL by *wt* and C70Δ*pheB* strains (Fig 6).

The accumulation of catechol inhibited the activity of C23O and probably also the HMS degrading enzyme (Fig 6B). In the case of the substrate mixture, catechol was not excreted into the growth medium, probably due to the contribution of the second *meta* pathway encoded by the *phe* genes (Fig 6C and 6D). SAL was degraded before PHE by both strains, probably as the result of bearing two salicylate hydroxylase genes. Therefore, we suppose that possessing two catechol degradation *meta* pathways gives an advantage to strain C70 in the environment, as contaminated ecosystems typically contain heterogeneous mixtures of organic compounds. Further research will be conducted to determine the substrate specificity of C23Os, the role of two redundant salicylate hydroxylase genes in strain C70 and the mechanism by which the PHE degradation pathway mitigates catechol toxicity during the degradation of SAL from the mixture of the substrates.

## Supporting information

S1 Fig A phylogenetic tree of catechol 2,3-dioxygenases. The phylogenetic tree is based on deduced amino acid sequences (307 aa) of the catechol 2,3-dioxygenases of strain C70 and reference strains from GenBank. The percentage of replicate trees in which the associated taxa clustered together in the bootstrap test (1000 replicates) is shown next to the branches.
(TIF)

S1 Table Bacterial strains and plasmids used in this study. (DOC)

S2 Table PCR primers used in this study. (DOCX)
