# Supplementary figures and images for: Strategy of Pseudomonas pseudoalcaligenes C70 for effective degradation of phenol and salicylate
Source: PLoS One. 2017 Mar 3;12(3):e0173180. doi: 10.1371/journal.pone.0173180 (PMC5336314; doi:10.1371/journal.pone.0173180)

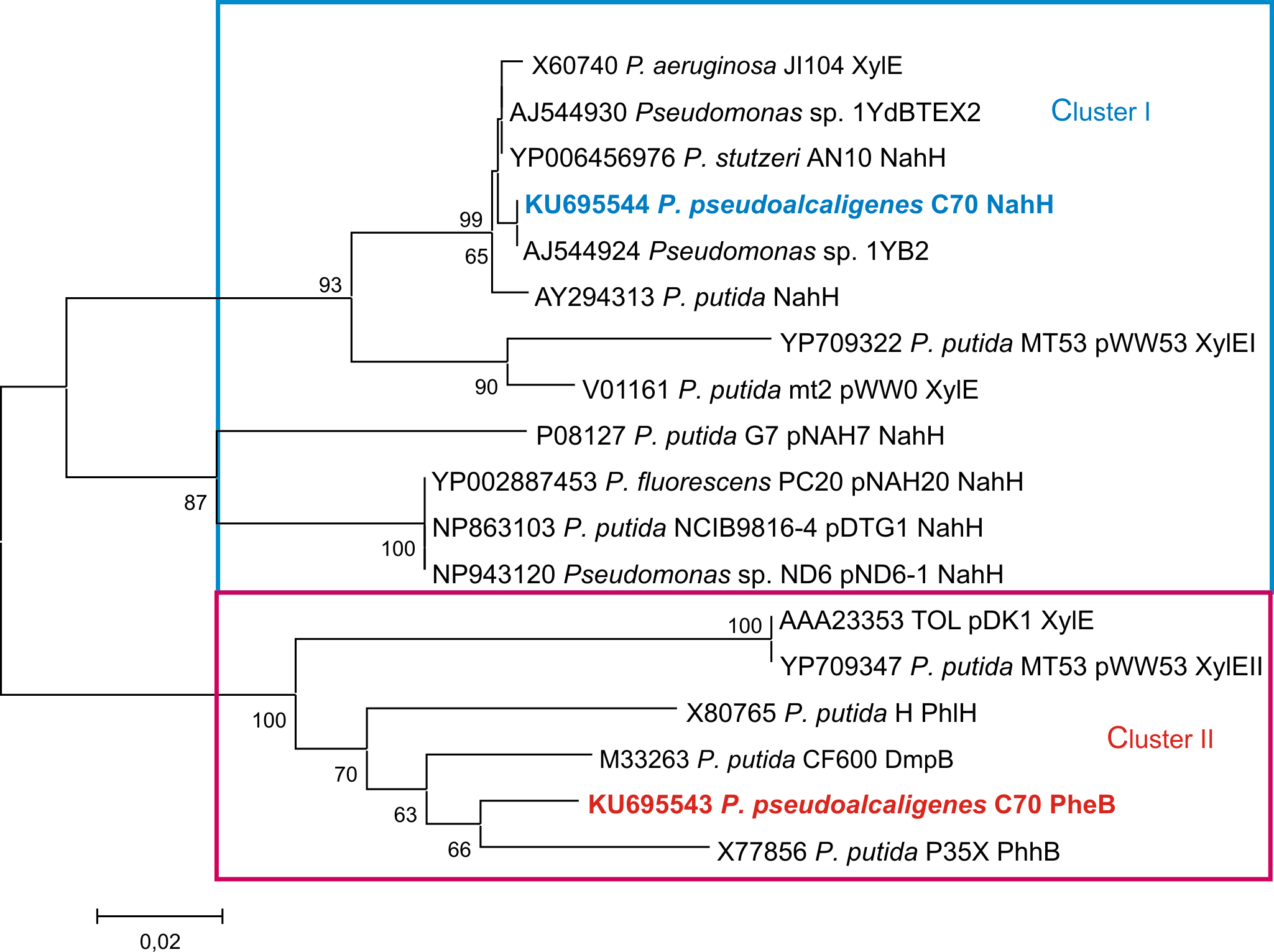

Supplement: S1 Fig — The phylogenetic tree is based on deduced amino acid sequences (307 aa) of the catechol 2,3-dioxygenases of strain C70 and reference strains from GenBank. The percentage of replicate trees in which the associated taxa clustered together in the bootstrap test (1000 replicates) is shown next to the branches. (TIF) [file pone.0173180.s001.tif]
