# Supplementary material for: Strategy of Pseudomonas pseudoalcaligenes C70 for effective degradation of phenol and salicylate
Source: PLoS One. 2017 Mar 3;12(3):e0173180. doi: 10.1371/journal.pone.0173180 (PMC5336314; doi:10.1371/journal.pone.0173180)
Supplement: S1 Table — (DOC) [file pone.0173180.s002.doc]

**S1 Table. Bacterial strains and plasmids used for this study.**

| **Strain or plasmid** | **Genotype or construction** | **Source or reference** |
| --- | --- | --- |
| ***P. pseudoalcaligenes*** |  |  |
| **C70** | Wild type |  |
| **C70∆*pheB*** | Mutant strain lacking *pheB* | this study |
| ***E.coli***  **DH5α** | *supE44* ∆*lacU169* (φ*80 lacZ* ∆*M15*) *recA1 endA1 hsdR17 thi-1 gyrA96 relA1* | Invitrogen, Thermo Fisher Scientific, USA |
| **HB101** | *subE44 subF58 hsdS3* (r_B_^-^ m_B_^-^) *recA13 ara-14 proA2 lacY1 galK2 rpsL20 xyl-5 mtl-1* |  |
| **CC118λpir** | ∆(*ara-leu*) *araD* ∆*lacX74 galE galK phoA20 thi-1 rpsE rpoB argE* (*Am*) *recA1* λ*pir* phage lysogen |  |
| **Plasmids**  **pTZ57R/T** | Cloning vector (Ap^r^) | Thermo Fisher Scientific, USA |
| **pUTmini-Tn5 Km2** | Delivery plasmid for mini-Tn*5* Km2 (Ap^r^ Km^r^) |  |
| **PBK-miniTn7-ΩGm** | pUC19-based delivery plasmid for miniTn7-ΩGm (Gm^r^, Ap^r^) |  |
| **pGP704 L** | Delivery plasmid for homologous recombination (Ap^r^) |  |
| **pRK2013** | Helper plasmid for conjugal transfer of pGP704 L (Km^r^) |  |
| **pTZ57R/C70nahH** | pTZ57R/T containing the PCR-amplified *nahH* gene of stain C70 from *nah* operon | this study |
| **pTZ57R/C70pheB** | pTZ57R/T containing the PCR-amplified *pheB* gene of stain C70 from *phe* operon | this study |
| **pTZ57R∆C70nahH::gm** | *nahH* of strain C70 (*nah* operon) in pTZ57R/T is interrupted with Gm^r^ gene from pBK-miniTn7-ΩGm by replacing *Aat*II and *Not*I-generated fragment from *nahH* by Gm^r^ gene | this study |
| **pTZ57R∆C70pheB::km** | *pheB* of strain C70 (*phe* operon) in pTZ57R/T is interrupted with Km^r^ gene from pUTmini-Tn*5* Km2 by replacing *Eco*130I generated fragment from *pheB* by Km^r^ gene | this study |
| **pGP704∆C70nahH::gm** | pGP704 L with *Kpn*I*-Pae*I fragment of ∆C70nahH::gm from pTZ57R∆C70nahH::gm in vector plasmid opened with the same restrictases | this study |
| **pGP704∆C70pheB::km** | pGP704 L with *Kpn*I*-Pae*I fragment of ∆C70pheB::km from pTZ57R∆C70pheB::km in vector plasmid opened with the same restrictases. | this study |

Ap^r^, ampicillin resistant,

Km^r^, kanamycin resistant,

Gm^r^, gentamycin reistant.

**References**

1. Vedler E, Heinaru E, Jutkina J, Viggor S, Koressaar T, Remm M, et al. *Limnobacter* spp. as newly detected phenol-degraders among Baltic Sea surface water bacteria characterised by comparative analysis of catabolic genes. Syst Appl Microbiol. 2013;36: 525-32.

2. Boyer HW, Roulland-Dussoix D. A complementation analysis of the restriction and modification of DNA in *Escherichia coli*. J Mol Biol. 1969;41: 459-72.

3. Herrero M, de Lorenzo V, Timmis KN. Transposon vectors containing non-antibiotic resistance selection markers for cloning and stable chromosomal insertion of foreign genes in Gram-negative bacteria. J Bacteriol. 1990;172: 6557-67.

4. de Lorenzo V, Herrero M, Jakubzik U, Timmis KN. Mini-Tn5 transposon derivatives for insertion mutagenesis, promoter probing, and chromosomal insertion of cloned DNA in Gram-Negative Eubacteria. J Bacteriol. 1990;172: 6568-72.

5. Koch B, Jensen LE, Nybroe O. A panel of Tn7-based vectors for insertion of the *gfp* marker gene or for delivery of cloned DNA into Gram-negative bacteria at a neutral chromosomal site. J Microbiol Methods. 2001;45: 187-95.

6. Pavel H, Forsman M, Shingler V. An aromatic effector specificity mutant of the transcriptional regulator DmpR overcomes the growth constraints of *Pseudomonas* sp. strain CF600 on para-substituted methylphenols. J Bacteriol. 1994;176: 7550-7.

7. Figurski DH, Helinski DR. Replication of an origin-containing derivative of plasmid RK2 dependent on a plasmid function provided in trans. Proc Natl Acad Sci U S A. 1979;76: 1648-52.
