# Supplementary material for: Strategy of Pseudomonas pseudoalcaligenes C70 for effective degradation of phenol and salicylate
Source: PLoS One. 2017 Mar 3;12(3):e0173180. doi: 10.1371/journal.pone.0173180 (PMC5336314; doi:10.1371/journal.pone.0173180)
Supplement: S2 Table — (DOCX) [file pone.0173180.s003.docx]

**S2 Table. PCR primers used in this study.**

| **Primer** | **Nucleotide sequence (5´→ 3´)** | **References** |
| --- | --- | --- |
| **C70aF**  **C70aR**  **C70bF**  **C70bR** | TGGAGCGGGATCTGACGGCA  CGGGATTGACCTCATTCACA  TCACCGAGGACCTGCTCAAC  CCGGGTTGACCTCGGCCAAG | In this study |
| **polAXhoylev**  **polAXhoall** | GGGGCAGAACGCCAAGTACG  TCTGCGCCAGGCTGTCCAT | [[1](#_ENREF_1)] |
| **C70a1F**  **C70a2R**  **C70b1F**  **C70b2R** | CAAACCAATAACAAGAGTTCG  ACGCCGTAGTTGAGTGCACC  CACCACCCTGATGCAAGGTCG  ATCTCGAAGGCTTCGGTG | In this study |
| **GmY**  **GmA** | CGAATTGACATAAGCCTGTTC  CGGCTTGAACGAATTGTTAG | [[1](#_ENREF_1)] |
| **KmSac**  **KmOc** | CAGGAGCTCGTTCGATTTATTCAACAAAGCC  TCGAGCAAGACGTTTCCC | [[2](#_ENREF_2)]  [[3](#_ENREF_3)] |

**References**

1. Sidorenko J, Jatsenko T, Saumaa S, Teras R, Tark-Dame M, Hõrak R, et al. Involvement of specialized DNA polymerases Pol II, Pol IV and DnaE2 in DNA replication in the absence of Pol I in *Pseudomonas putida*. Mutat Res 2011;714: 63-77.

2. Hõrak R, Ilves H, Pruunsild P, Kuljus M, Kivisaar M. The ColR-ColS two-component signal transduction system is involved in regulation of Tn4652 transposition in *Pseudomonas putida* under starvation conditions. Mol Microbiol. 2004;54: 795-807.

3. Saumaa S, Tarassova K, Tark M, Tover A, Tegova R, Kivisaar M. Involvement of DNA mismatch repair in stationary phase mutagenesis during prolonged starvation of *Pseudomonas putida*. DNA Repair 2006;5: 505-14.
